# Publisher Correction: High power surface emitting terahertz laser with hybrid second- and fourth-order Bragg gratings

**DOI:** 10.1038/s41467-018-04400-8

**Published:** 2018-05-14

**Authors:** Yuan Jin, Liang Gao, Ji Chen, Chongzhao Wu, John L. Reno, Sushil Kumar

**Affiliations:** 1 Department of Electrical and Computer Engineering, Lehigh University, Bethlehem, PA 18015 USA; 2 Sandia National Laboratories, Center of Integrated Nanotechnologies, MS 1303, Albuquerque, NM 87185 USA

Correction to: *Nature Communications* 10.1038/s41467-018-03697-9, published online 11 April 2018

The original PDF version of this Article contained an error in Equation 1. The ‘Λ’ was missing from the denominator, and incorrectly read:

$$n\frac{{2{\mathrm{\pi }}}}{{}} = k_{\mathrm{i}} + k_{\mathrm{d}}\,{\mathrm{sin}}\,(\theta_{\mathrm{d}}).$$

The correct form of Equation 1 is:

$$n\frac{{2{\mathrm{\pi }}}}{\Lambda } = k_{\mathrm{i}} + k_{\mathrm{d}}\,{\mathrm{sin}}\,(\theta_{\mathrm{d}}).$$

This has been corrected in the PDF version of the Article. The HTML version was correct from the time of publication.

