# Supplementary material for: High power surface emitting terahertz laser with hybrid second- and fourth-order Bragg gratings
Source: Nat Commun. 2018 Apr 11;9:1407. doi: 10.1038/s41467-018-03697-9 (PMC5895695; doi:10.1038/s41467-018-03697-9)
Supplement: Supplementary file 1 — Supplementary Information(PDF 2087 kb) [file 41467_2018_3697_MOESM1_ESM.pdf]

Supplementary Information

High power surface emitting terahertz laser with hybrid second- and fourth-order Bragg gratings

Jin et al.

Supplementary Note 1. Coupling coefficient for the hybrid distributed-feedback (DFB) gratings

In an optical cavity with Bragg gratings for DFB, the coupling coefficient κ is a key factor to describe the coupling strength of the forward and backward traveling guided waves and should not ideally be less than the reciprocal of the cavity's length for a uniform field distribution as determined from coupled-mode theory [1]. For a cavity with one-dimensional periodic gratings, the eigen-frequencies $\omega_\pm$ of the two band-edge modes are related to the complex coupling coefficient through the following relation [2]

$$\kappa + i\kappa_i = \frac{n_g}{2c}(\omega_+ - \omega_-) \tag{1}$$

where c is the speed of light in vacuum and n_g denotes the group refractive index. Using simulation results of the hybrid second- and fourth-order DFB grating from Fig. 2(b) with $d/\Lambda = 3/8$, $\Lambda = 27$ μm and assuming no waveguide dispersion, Supplementary Equation (1) leads to $\kappa \sim 11.7$ cm^{-1}. This relatively large value translates to a determination of the optimum length of laser cavity to $\sim$ 1 mm (not including the length of bonding pads at the longitudinal ends of the cavity). A slightly longer length of 1.3 mm was chosen for implementation to minimize the optical losses due to longitudinally absorbing boundaries.

Supplementary Note 2. Finite-element modeling of terahertz QCL cavities with hybrid DFB gratings

Finite-element (FEM) simulations were performed to comprehensively understand the characteristics of hybrid second- and fourth-order DFB scheme. Supplementary Figure 1(b) shows electric-field distribution as well as energy-density profile for the resonant band-edge modes of the cavities with hybrid DFB scheme, along the entire length ($\sim$ 1.3 mm) of the cavity with gratings implemented in the top metal cladding. Energy density is estimated along the vertical center of the cavity from 2D simulations that effectively models cavities of infinite width. The eigenmode spectra and electric-field distributions for the cavities show non-uniform envelope shapes, which gradually decay from the center to the end-facets of cavity. This is due to the typical DFB action that couples the propagating waves along the

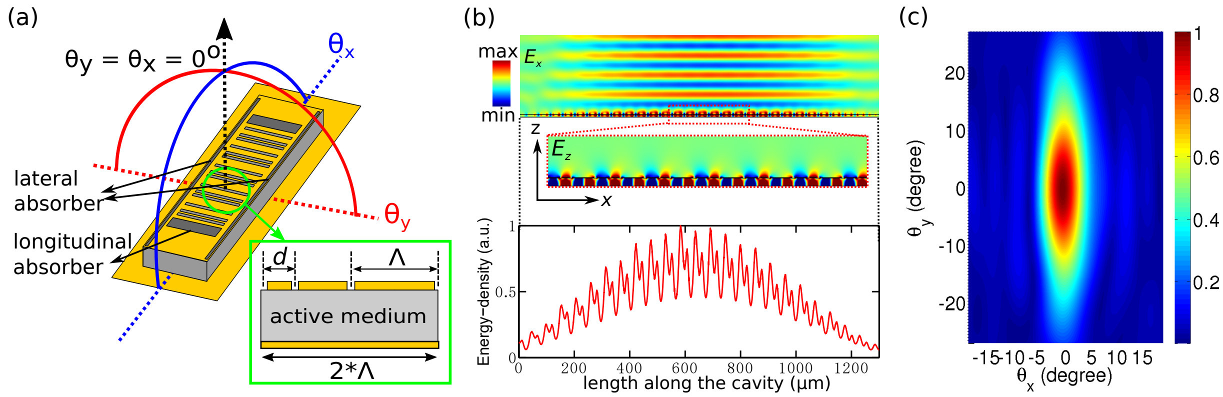

Supplementary Figure 1: **Results from finite-element modeling of QCL cavity with hybrid DFB gratings** (a) The schematic on left shows the QCL's metallic-cavity with hybrid DFBs grating implemented in top metal cladding indicating the orientation of QCLs and definition of angles. (b) The figures shows the electric-fields for the eigenmode with the lowest-loss (upper band-edge mode) calculated by FEM simulations of parallel-plate metallic cavities, and the energy-density in the cavity along the length of $\sim$ 1.3 mm long metallic cavity (in x direction). (c) Full-wave 3D simulation result of the far-field radiation pattern of a 1.5 mm $\times$ 200 μm $\times$ 10 μm cavity with hybrid DFB gratings. The full-width half-maximum (FWHM) is $\sim 7.5° \times 30°$.

length of cavity.

To further validate the experimental results of Fig. 4, full-wave 3D FEM simulations were carried out for terahertz QCL cavities with the hybrid DFB scheme. Supplementary Figure 1(c) shows the computed far-field radiation pattern for a band-edge DFB mode of a terahertz QCL cavity implemented with the hybrid DFB gratings in the top metal cladding. In order to set up a far-field calculation, a far-field domain node is implemented that is a single closed surface surrounding all radiating apertures in the cavity. Simulated 3D beam pattern demonstrates single-lobed beam for the upper band-edge mode with narrow divergence in the θ_x direction and large divergence in the θ_y direction, which corresponds with the experimental results shown in Fig. 4. The shape and FWHM of the computed radiation pattern is in close agreement but slightly larger than that of the measured results. The difference is likely due to the relative simplification of implemented 3D model, which does not account for the sloped sidewall profiles of the cavities.

It is also noticeable that the lower band edge mode (antisymmetric mode) of the hybrid DFB scheme would also show a single-lobed far field beam pattern due to a constructive interference along the entire length of cavity. Electric-field distribution and the computed far-field radiation patterns from full-wave 3D FEM simulations of lower band edge mode

is shown in Supplementary Figure 2, which has a similar size of FWHM with that of the symmetric mode.

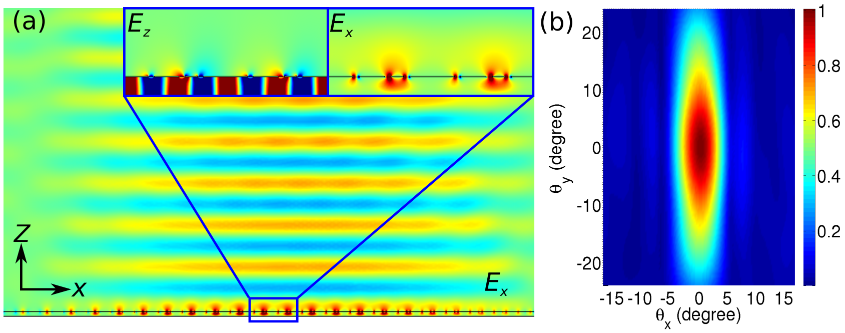

Supplementary Figure 2: **Finite-element simulation of lower-band edge mode (anti-symmetric mode) of hybrid DFB** (a) This figure shows the electric field of anti-symmetric mode calculated by FEM simulations, which demonstrates a constructive interference along the length of laser cavity. (b) Full-wave 3D simulation results of the far-field radiation pattern of the lower band-edge mode with hybrid DFB gratings.($\Lambda = 27$ μm, $d/\Lambda = 0.375$)

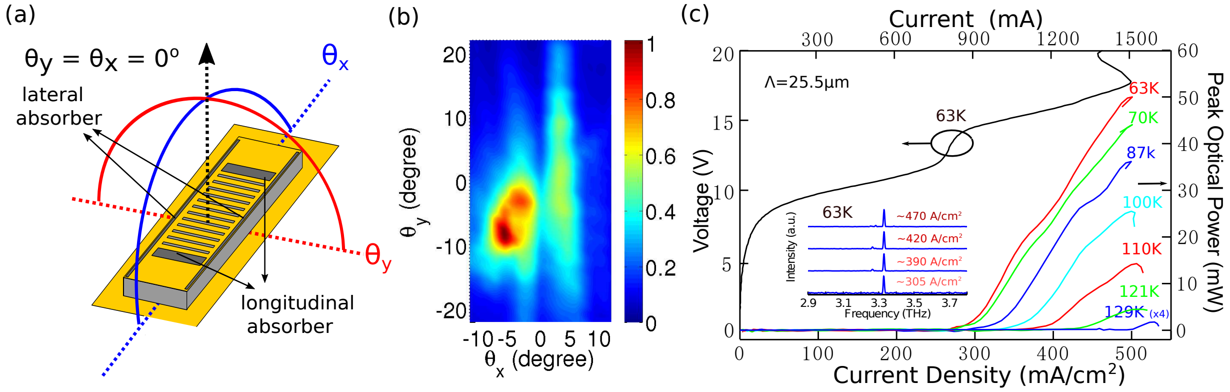

Supplementary Figure 3: **Lasing characteristics of a terahertz QCL with conventional second-order DFB** (a) A schematic with indication of orientation of the QCL and definition of angles of the QCL's metallic-cavity with second-order DFB grating implemented in top metal cladding. (b) Measured far-field radiation-pattern of the QCL that is fabricated from the same MBE wafer as that for the the QCL whose data is presented in Fig. 4. A two-lobed beam is detected. (c) Experimental light-current-voltage characteristics of the representative QCL with second-order DFB grating of period 25.5 μm and cavity dimensions 1.55 mm × 200 μm × 10μm at different heat-sink temperatures. Insets shows lasing spectra at different bias. The QCL emits predominantly in single-mode at $\lambda = 90$ μm (3.33 THz).

Supplementary Note 3. Experimental results for a QCL with conventional second-order gratings

To better illustrate the advantages of the hybrid DFB scheme over conventional second-order DFB for terahertz QCLs, Supplementary Figure 3 shows the lasing characteristics of a representative QCL with second-order DFB gratings that was fabricated with the same active medium by standard lithography to pattern gratings with similar duty-cycles in the top-metal cladding, and with similar cavity dimensions as that of the hybrid DFB QCLs whose data is reported in Fig. 4. Using the wavelength equation of $\lambda = 2n_{\text{eff}}\Lambda/p$ ($p = 2$) for second-order DFB, the grating period was chosen as 25.5 μm so that lasing mode could be excited in the frequency range of $3.3 - 3.4$ THz, which is the range at which peak gain is realized from the active medium. The measured peak-optical power is $\sim$ 50 mW in pulsed mode of operation at 63 K and the wall-plug efficiency is $\sim$ 0.18 %. A slope-efficiency of 81 mW A^{-1} is also estimated, which is lesser by more than an order of magnitude when compared to the QCL with hybrid DFB gratings due to the poor radiative efficiency from the conventional gratings.

It should be noted that an exact quantitative comparison of hybrid DFB with conventional second-order DFB is difficult. Absorbing boundaries are desired to eliminate reflections from the facets in order to achieve robust single-mode operation. For conventional second-order DFB with short cavities, the out-coupling efficiency can be considerably affected by the boundary conditions [3]. However, for relatively long cavities ($>$ 2 mm), the effect of boundary conditions on the surface-loss becomes negligible since the intensity of the standing-wave in the cavity vanishes near the facets for the lower band-edge mode.

Supplementary Note 4. Experimental results for a Fabry-Pérot cavity QCL

Supplementary Figure 4 shows $L - I$ characteristics of a representative Fabry-Pérot QCL that was fabricated with same active medium as that used for DFB QCLs. This QCL operated up to a heat-sink temperature $\sim$ 137 K with J_{th} $\sim$ 280 A cm^{-2} at 62 K. The shown Fabry-Pérot QCL shows a similar slope efficiency (for emission from one facet, collected with a Winston cone) as that of standard second order DFB (for emission collected in surface-normal direction, without a Winston cone), which is $\sim$ 67 mW A^{-1}.

The threshold current densities and slope efficiency of Fabry-Pérot device are highly consistent with those of conventional 2nd order DFB, which is expected. For the conventional second order DFB QCLs and Fabry-Pérot QCLs, the out-coupling efficiencies are expected to be quite low. Therefore, the optical gain near threshold is dominated by waveguide loss in both cases, which should result in similar threshold current-densities Comparing these threshold current densities to that for the QCL with hybrid DFB gratings reveals that the threshold density is much higher for the latter, which is primarily due to a higher optical loss in the cavity due to increased radiative outcoupling.

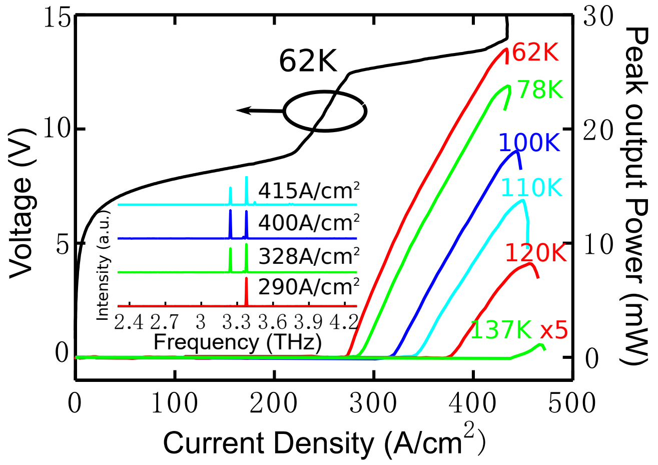

Supplementary Figure 4: **Lasing characteristics of a Fabry-Pérot terahertz QCLs**. $L-I$ and $I-V$ characteristics from a 1.55 mm long and 150 µm wide laser biased in pulsed operation are shown. The corresponding insets show laser spectra measured at 62 K. The QCLs radiation frequency was centered around 3.3 THz.

Supplementary Note 5. Comparison between hybrid DFB and graded photonic heterostructures

Before the demonstration of this work, graded periodicity of second-order gratings (GPH) was used as an effective method to improve the power output level of single-mode surface-emitting (SE) terahertz QCLs [4], which led to the previous highest power for single mode terahertz QCLs. In this section, the performance of GPH and hybrid DFB is compared through FEM simulations.

For resonant phonon depopulation based active regions, the peak photon density in the lasing mode of terahertz QCLs could be approximated as a constant, which indicates that

the peak-value of electric energy density inside laser cavity should have an equal intensity across different QCLs for a fixed amount of current-density biased above threshold for a given active region. Thus, when theoretically comparing hybrid DFB with GPH DFB, it is necessary to normalize the electric energy density inside the active medium. Supplementary Figure 5 shows the normalized time average of electric energy-density profile for the resonant band-edge modes of the cavities with hybrid second- and fourth- order DFB and graded photonic heterostructure respectively. For hybrid DFB $\Lambda = 27$ μm, $d/\Lambda = 0.375$, for GPH, $a_0 = 27$ μm, the resonant modes of both two designs are around 3.4 THz. The length of cavities for both cases are selected according to the reported structure in this work and that of Ref.[4]. Energy-density is calculated along the cavitys vertical center (5 μm) from 2D simulations (that effectively model cavities of infinite width). Both hybrid DFB and GPH show non-uniform envelope shapes, which gradually decrease from the center to the end-facets of cavity and provide indication of typical DFB action due to coupling of propagating waves along the length of cavity as per the corresponding schematics.

However, it is noted that a considerable large non-uniformity of electric field distribution could be observed from Supplementary Figure 5.(b) for the presented GPH design in Ref.[4], the full width half maximum of electric energy density profile is only $\sim$ 180 μm, which indicates that the rest part of laser cavity is not fully utilized to provide power output. In contrast, the FWHM of electric energy density profile of hybrid-DFB is as large as $\sim$ 620 μm, hence the stimulated emission could be generated in a large fraction of laser cavity, which is significantly strengthened (by approximately three times) than that of the GPH cavity. Simulation of further increase in length of cavities of GPH shows a similar electric-field distribution with similar FWHM of electric energy density profile, which verifies that the large non-uniformity of electric field distribution of GPH is not determined by the length of laser cavities but is due to its specific DFB coupling of the GPH itself. To summarize, due to the internal defect of GPH, a large non-uniformity in the electric field distribution of its resonant mode, would make it difficult to further enhance the radiative efficiency significantly in GPH based SE terahertz lasers due to the small volume of usable optically pumped active region compared to that of the hybrid DFB.

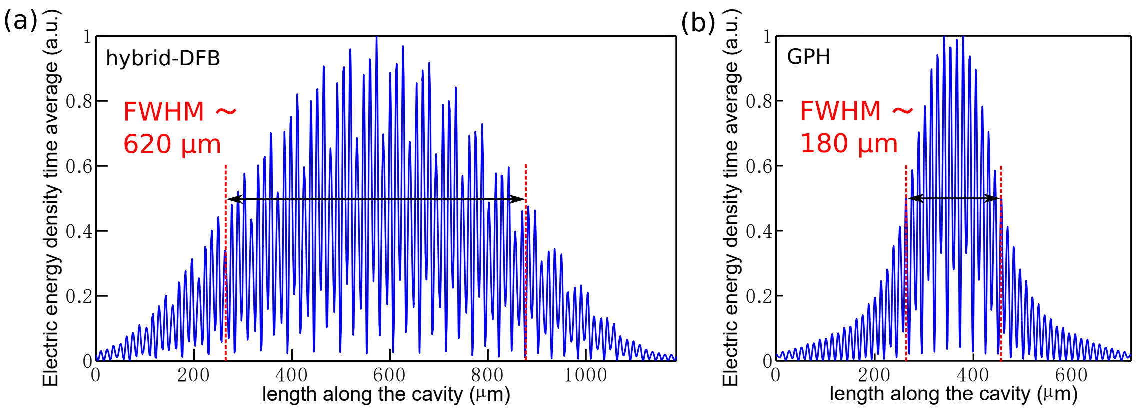

Supplementary Figure 5: Time average of electric energy-density profiles along the length of the metallic cavity (in x direction) of THz-QCLs for the resonant mode of (a) hybrid DFB and (b) graded photonics heterostructure (GPH).

Supplementary Note 6. FEM modeling of terahertz QCL cavities with fourth-order DFB gratings

FEM simulations and analysis were also carried out for fourth order DFB gratings to verify if off-normal directional beam profiles exist. For a terahertz QCL with fourth order gratings, $2\pi/\Lambda = k_i/2$ ($\Lambda = 2\lambda_w$, λ_w is the wavelength inside active medium) and n_w, representing the refractive index of waveguide, is about $\sim$ 3.6 . Eq. (1) of the main paper shows that the 2nd order and 4th order diffraction of fourth order DFB gratings would result into vertical reflection ($\theta_d = 0$, $k_d = k_i/n_w$) and backward reflection ($\theta_d = \pi/2$, $k_d = k_i$) respectively. It is assumed that $k_d = k_i/n_w$ if any off-normal-direction lobes are existing. Eq.(1) variants to $nk_i/2 = k_i + k_i \sin(\theta_d)/3.6$. Considering odd-order diffractions that $n =$ 1 and 3, $|\sin(\theta_d)|$ should be $\sim$ 1.8, no viable solution could be found for phase-matching. This fact reveals that there does not have any off-normal-direction lobes for terahertz QCLs with fourth order DFB gratings.

Supplementary Figure 6 shows the radiative surface losses of the resonant modes, their frequencies and electric-field features for the band-edge modes for finite-length cavities with fourth-order Bragg gratings implemented in the top metal layers. The lower and upper band-edge modes have similar characteristics of electric-field distribution as standard second-order DFB, which respectively show two-lobe and single-lobe beam profiles, as well as low out-coupling and high out-coupling efficiency respectively. Supplementary Figure 6(c) verifies that there does not exist off-normal direction beam branch in the near- or far-field of fourth

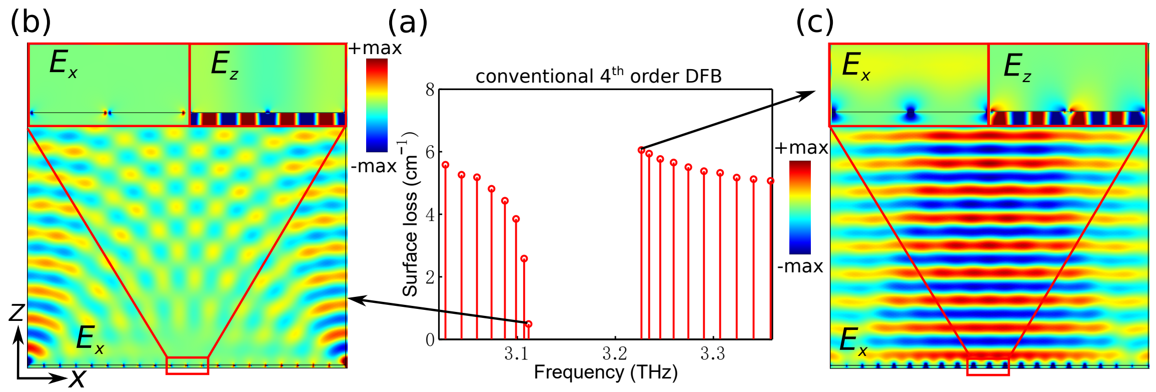

Supplementary Figure 6: **FEM modelling of terahertz QCLs with fouth-order gratings** (a) Mode-spectrum for a infinitely wide cavity with fourth order Bragg gratings ($\Lambda = 54$ µm, slit-width $\sim$ 4 µm). (b),(c) these figures shows the electric-fields for the eigenmodes of upper and lower band-edge calculated by FEM simulations of parallel-plate metallic cavities, which respectively reveal two-lobed beam behavior and single-lobed beam profile for antisymmetric and symmetric mode.

order DFB. It is also noted that the surface loss of upper band-edge mode (symmetric mode) of fourth-order DFB is much lower than that of second-order DFB, which could be explained by noting that half of the slits on metal cladding layer of second-order DFB are closed. That is, the number of effective radiating apertures is halved.

Supplementary Note 7. Relation between the slope-efficiency and the outcoupling efficiency

The power out-coupled from a surface-emitting QCL with uniform distribution of electromagnetic field in the cavity can be expressed as

$$P_{out} = \hbar\omega_0 n_{ph}^{st} \alpha_s \frac{c}{n_r} = \frac{N_p \hbar\omega_0}{|e|} \frac{\alpha_s}{\alpha_w + \alpha_r} [(I - I_{th})\chi] \qquad (2)$$

In Supplementary Equation (2), N_p is the number of repeated modules in the QCL, c/n_r represents the speed of light inside the cavity, α_s, α_r and α_w are optical losses due to surface-radiation, overall radiation (including that from facets), and due to optical losses in the waveguide (due to free-carrier and intersubband absorption) respectively, n_{ph}^{st} represents the stimulated photon number in the lasing mode above threshold, which is proportional to $(I - I_{th})\chi$, I is electronic current (in the direction of electron transport) flowing through the QCL, I_{th} is the threshold current, and χ is the internal differential efficiency of the QCL

superlattice. For a given type of active medium (such as the one based on resonant-phonon depopulation here) and at a certain given frequency, the internal differential efficiency χ and the waveguide loss α_w are relatively constant across small variations in the superlattice designs, and also do not depend significantly on the DFB design of the cavity. Hence, $\alpha_s/(\alpha_w + \alpha_r)$, defined as out-coupling efficiency [5], directly corresponds to the level of output power from the QCL. Also, by differentiating Supplementary Equation (2) with current I, the slope efficiency dP_{out}/dI_{out} becomes

$$\frac{dP_{out}}{dI} = \frac{N_p \hbar\omega_0}{|e|} \frac{\alpha_e}{\alpha_e + \alpha_w} \chi \tag{3}$$

which suggests that the slope-efficiency for a given internal differential efficiency is an intuitive indicator of the out-coupling efficiency of the QCL.

Supplementary References

[1] Kogelnik, H. & Shank, C. Coupled-wave theory of distributed feedback lasers. *J. Appl. Phys.* **43**, 2327–2335 (1972).

[2] Wienold, M. *et al.* Lateral distributed-feedback gratings for single-mode, high-power terahertz quantum-cascade lasers. *Opt. Express* **20**, 11207–11217 (2012).

[3] Kumar, S. *et al.* Surface-emitting distributed feedback terahertz quantum-cascade lasers in metal-metal waveguides. *Opt. Express* **15**, 113 (2007).

[4] Xu, G. *et al.* Efficient power extraction in surface-emitting semiconductor lasers using graded photonic heterostructures. *Nature. Comm.* **3**, 952 (2012).

[5] Sigler, C. *et al.* Design for high-power, single-lobe, grating-surface-emitting quantum cascade lasers enabled by plasmon-enhanced absorption of antisymmetric modes. *Appl. Phys. Lett.* **104**, 131108 (2014).
